# Editorial: Insights in nanobiotechnology 2022/2023: novel developments, current challenges, and future perspectives

**DOI:** 10.3389/fbioe.2023.1331760

**Published:** 2023-11-10

**Authors:** Gianni Ciofani, Marco P. Monopoli

**Affiliations:** ^1^ Istituto Italiano di Tecnologia, Smart Bio-Interfaces, Pontedera, Italy; ^2^ RCSI University of Medicine and Health Studies, Chemistry Department, Dublin, Ireland

**Keywords:** stem cells, liposomes, extracellular vesicles (EVs), nanoparticles, immunology, pandemic (COVID-19), neurosurgery

The Research Topic “*Insights in nanobiotechnology 2022/2023: novel developments, current challenges, and future perspectives*” is an annual Research Topic featuring peer reviewed reviews, opinion papers, and research articles that looks to explore new insights, novel developments, current challenges, latest discoveries, recent advances, and future perspectives in the field of Nanobiotechnology. Among the 10 peer reviewed manuscripts published in the framework of this Research Topic, we have four Original Researches, three Reviews, one Mini-Review, and two Opinions.

Testa et al. report on a whole transcriptomic analysis of stem cells cultured on microfabricated scaffolds, named “Nichoids”, showing as stemness is preserved with respect to traditional 2D cultures, highlighting the important implication this culture approach owns for different applications in biomedicine.

Moving to the fields of nanoparticles for theranostic applications, Ota el al. present an innovative strategy for liposome preparation, comparing its performance, in terms of encapsulation efficiency, drug loading, lamellarity, and user-friendliness with a commonly used microfluidic device.

Jakl et al. show a novel approach for large-scale manufacturing of small extracellular vesicles (EVs) from bone marrow-derived mesenchymal stromal cells. EVs are membrane-surrounded nanostructures secreted ubiquitously by cells, *i.e.*, “cellular nanoparticles” mediating communication among cells and containing hundreds of molecules, including miRNA, proteins, DNA, and lipids. These molecules work synergistically to activate multiple cells, and thus EV-based therapy is considered as the next-generation of stem cell therapy.

The last Original Research, from Rudi et al. is instead focused on the effects citrate-stabilized gold and silver nanoparticles on cultures of microalgae. These nanomaterials resulted to be a stress factor for red microalga Porphyridium cruentum, causing significant changes in both biotechnological and biomass safety parameters, and leading to an enhanced lipid accumulation and reduced malondialdehyde values in the biomass.

Coming to reviews, Mobeen et al. depict an overview of the emerging role of nanotechnology in immunology, highlighting novel theranostic immunological applications of nanomedicine. Yang and Tel, instead, presents recent advancements in global and local signal generators, highlighting their applications in studying temporal and spatial cellular signalling activity.

The third Review in this Research Topic, from Lin et al., highlights how nanoplatforms can be tailored for targeted delivery to dendritic cells, thus inducing immune tolerance: this approach envisions great perspectives in the treatment of autoimmune diseases, organ transplantation, and allergic diseases.

In the mini-review of Porello and Cellesi, the authors provide a nice overview of the state-of-the-art methods for intracellular protein delivery to mammalian cells, highlighting current challenges, new developments, and future research opportunities.

The Opinion paper of Limongi and Susa is focused on one of the major recent challenge faced by the humankind, *i.e.*, the COVID-19 pandemic. The Authors focused their attention on how much nanobiotechnology contributed and still is contributing to the development of safe and efficient solutions to prevent, diagnose, and treat COVID-19, and any similar infection or pathology.

Lam et al. describes important translational issues in Nanobiotechnology that can be used as theranostics in neurosurgical oncology, highlighting recent advancements in the application of nanoplatforms for fluorescence image-guided brain tumor resection and treatment.

Concluding, we hope this Research Topic could have provided useful cues and insights to the Readers, shedding lights on the most recent development in the application of nanotechnology to biology and human healthcare.

